# Retraction: Comparative proteomic analysis reveals the regulatory network of the *veA* gene during asexual and sexual spore development of *Aspergillus cristatus*

**DOI:** 10.1042/BSR-20180067_RET

**Published:** 2021-04-27

**Keywords:** ascospore, Aspergillus cristatus, conidia, proteomics, veA gene

This Retraction follows an Expression of Concern relating to this article previously published by Portland Press.

This article is being retracted from *Bioscience Reports* at the request of the authors following receipt of a notification from a reader, alerting the authors to the possibility of spliced blots in Figure 5A. The authors have been unable to replicate the results and wish to retract the article. The Editor-in-Chief and Editorial Board agree with the retraction.

